# Design of Microdisk-Shaped Ge on Si Photodetector with Recess Structure for Refractive-Index Sensing

**DOI:** 10.3390/s19235253

**Published:** 2019-11-29

**Authors:** Dongjun Seo, Chang-Soo Park, Young Min Song

**Affiliations:** School of Electrical Engineering and Computer Science, Gwangju Institute of Science and Technology (GIST), 123 Cheomdangwagi-ro, Buk-gu, Gwangju 61005, Korea

**Keywords:** silicon on insulator, refractive index sensor, germanium photodetector, disk resonator

## Abstract

In this paper, we introduce a disk-shaped Ge-on-Si photodetector for refractive-index difference sensing at an operating wavelength of 1550 nm. For the implementation of a small-scale sensor, a Ge layer was formed on top of a Si layer to increase the absorption coefficient at the expense of the light-detection area. Additionally, the sensor had a ring waveguide structure along the edge of the disk formed by a recess into the inner part of the disk. This increased the interaction between the dominant optical mode traveling along the edge waveguide and the refractive index of the cladding material to be sensed, and conclusively increased detection sensitivity. The simulation results show that the proposed sensor exhibited a detection sensitivity of >50 nm/RIU (Refractive Index Unit), a quality factor of approximately 3000, and a minimum detectable refractive index change of 0.95 × 10^−2^ RIU with a small disk radius of 3 μm. This corresponds to 1.67 times the sensitivity without a recess (>30 nm/RIU).

## 1. Introduction

Optical resonators are widely used in the environmental and industrial fields, for example, as sensing devices to measure the refractive index of gases and fluids, due to their advantage of immunity to electromagnetic interference. When the optical mode propagates through the resonator, the resonant wavelength is influenced by the refractive index of the material surrounded by the resonator. Various structures to detect changes in the resonant wavelength have been introduced, such as disk resonators [1,2], Bragg gratings [3], photonic crystals [4,5], Mach–Zehnder interferometers [6], and ring resonators [7,8]. Recently, as applications have expanded to the biomedical field, many efforts have been made to reduce the sensor size. Among them is an optical sensor using silicon photonics technology that allows sensing parts, such as light sources, sensors, detectors, and read-out electronics, to be integrated as a single-chip [9] integrated photonic and electronic device by using a silicon-on-insulator (SOI) platform [10], providing complementary metal–oxide–semiconductor (CMOS) compatibility and well-commercialized silicon-based fabrication. Therefore, SOI-based photonic sensors are attractive for future applications as lab-on-a-chip (LoC) systems and can also be multiplexed in a sensor array and bonded with micro- or nanofluidic structures at a low cost [11].

As photodetector-type sensors, Si disk photodetectors [12] and Ge PIN (positive-intrinsic-negative) photodetectors [13] have been reported as label-free refractive-index sensors. In this structure, the absorption coefficient of the material determines the amount of absorbed light per unit volume. Thus, an Si disk photodetector requires a relatively large disk area owing to the low absorption coefficient at 1550 nm operation (it has the advantage of using commercially available optical devices from the telecommunications market). As a result, the detected signal is lower than that of Ge with the same volume. On the contrary, a Ge photodetector has a high absorption peak, and a small-area (few square micrometers) design is possible. Furthermore, a disk-type Ge photodetector is free from the sensitivity degradation caused by dark currents because it is based on resonant-wavelength detection, not intensity detection. A simple Ge PIN structure also has a low quality factor, showing poor sensing ability due to the low interaction between refractive index and the light inside the PIN structure.

In this paper, we focus on the disk-shaped resonator that has a suitable structure to implement a small size and introduce two types of disk-shaped Ge on Si photodetector sensors: purely disk-shaped, and disk-shaped with a recess structure. A Ge layer on top of the Si layer increases the absorbed power, and thus decreases the size of the sensor. It was also expected that the recess structure of the Si layer forms a waveguide along the edge of the disk. Thus, it generates the dominant mode along the waveguide, and increases the interaction between the refractive index of the cladding material and the propagating optical modes along the circular waveguide, enhancing the sensitivity. For performance evaluation, sensor characteristics were simulated using the Finite-Difference Time-Domain (FDTD) method. The resonance characteristics depend on the propagation mode, mode loss, and the interacting area between the mode and the refractive index of the cladding material. These parameters are also related to geometrical parameters such as disk thickness, radius, and size. All simulations were conducted with the FDTD and MODE solutions of Lumerical Inc., Vancouver, BC, Canada.

## 2. Background and Disk-Shaped Photodetector Structure

**Figure 1:**
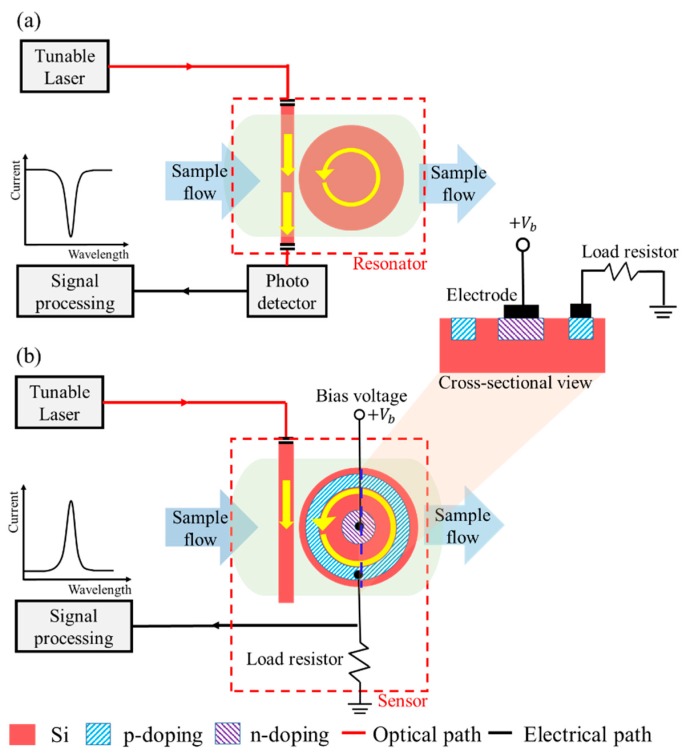
Schematic diagrams of disk-shaped sensors: (**a**) separate form; (**b**) combined form.

Figure 1 shows two types of disk sensor. The sensor in Figure 1a shows a disk-type resonator with a bar for input light; for signal detection, a separate photodetector is required. On the contrary, in Figure 1b, the photodetector is combined with the resonator by a fabrication process. The input light is excited to the bar (straight waveguide) and coupled to the disk. The coupled light propagates inside the disk, and the resonant mode is not back-coupled to the bar. Then, the output light is electrically converted by the photodetector. The resonant mode is influenced by the refractive index of the material (gases or fluids; hereafter, cladding material) surrounding the disk. Therefore, the resonant wavelength varies due to changes in the refractive index. Finally, by scanning the wavelength of the optical source and detecting the peak current, we can identify the material by the refractive index. In Figure 1b, we can see that the PIN photodetector was grown on top of the bottom disk in a combined form, and a small-size fabrication process was possible. With the advantage of compatibility with the CMOS process, an electrical amplifier could also be integrated as a one-chip system.

The performance of a disk resonator-type sensor can be evaluated with regard to three parameters: sensitivity (*S*), quality factor (*Q*), and intrinsic limit of detection (*ILOD*). *S* is defined as the degree of resonant-wavelength shift with respect to a change in the refractive index of the cladding material, and can be written as [14]:

(1) $$S=\frac{\Delta \lambda_{res}}{\Delta n_{c}}$$

where *λ_res_* and *n_c_* are the resonant wavelength and refractive index of the cladding material, respectively. The unit of *S* is nm/RIU, and RIU stands for Refractive Index Unit. Because the target material flows in the cladding of the sensor structure, the refractive-index change (Δ*n_c_*) in the cladding depends on the material. In a disk resonator, *λ_res_* is a function of the effective refractive index (*n_eff_*) and the radius of the disk. Therefore, the resonant wavelength of a resonator with a two-layer disk (Ge and Si) structure depends on the radii of the Ge and Si disks. *Q* represents the resolution of the sensor and is determined by the dominant optical mode propagating along the disk until the mode energy decreases to 1/e of the maximal value. It can be written and approximated as [14]:

(2) $$Q=\frac{2\pi n_{g}\times 4.34}{\alpha \lambda_{res}}\approx \frac{\lambda_{res}}{\Delta \lambda_{3dB}}$$

where *n_g_*, *α*, and Δ*λ_3dB_* are the group index, loss in the resonator (dB/m), and full width at half maximum of the resonance, respectively. As can be seen from Equation (2), the quality factor indicates the sharpness of the resonance, showing the detector’s ability to distinguish resonance peaks. In general, to evaluate a minimum detectable refractive-index change, the intrinsic characteristics of the resonator and those of the measurement system should be measured. However, the *ILOD* is frequently considered to compare the sensing performance of resonators, and is defined as [14]:

(3) $$ILOD=\frac{\lambda_{res}}{QS}$$

As shown in Equation (3), the *ILOD* is inversely proportional to *S*. Therefore, the *ILOD* can be improved by increasing *S*, which is also increased by increasing the interaction between the optical modes propagating inside the disk and the cladding material.

**Figure 2:**
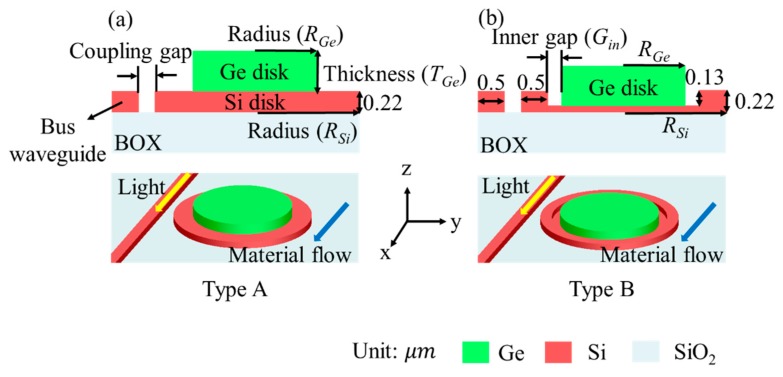
Disk-shaped sensors with and without recess structure (upper: side view; lower: top view): (**a**) Type A; (**b**) Type B.

The two proposed types of disk-shaped sensor, namely, Types A and B, are shown in Figure 2. Both types have the combined form of a resonator and a photodetector. However, for performance comparison, the characteristics of the photodetector were not considered. Type A was a purely disk-shaped structure, and Type B was a disk-shaped structure with a recess on top of the Si disk surface. These two structures were based on a 220 nm SOI platform with a 2 μm thick buried oxide (BOX) layer that is conventionally used for foundry services. The width and height of the bus waveguide were set to 500 and 220 nm, respectively, to meet the single-mode condition at a wavelength of 1550 nm [15]. The etch depth of the recess in Type B was set to 130 nm, taking into account both the light confinement inside the ridge and the light absorption into the Ge disk layer. At a shallow etch depth, the amount of light propagating inside the Si disk was reduced because light was more easily coupled to the Ge disk. On the contrary, a deep etch depth obstructed light from being coupled to the Ge disk and resulted in the low responsivity of the photodetector.

**Figure 3:**
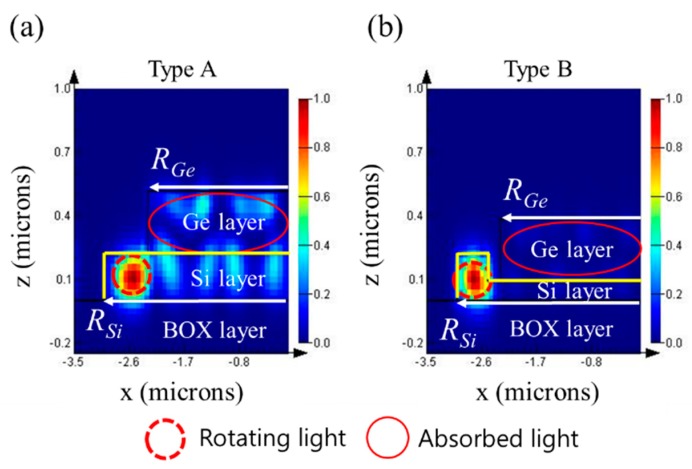
Normalized electric-field intensities of modes propagating along disk at 1550 nm wavelength for two structures: (**a**) Type A; (**b**) Type B.

Figure 3 shows the mode intensity propagating inside the disk after being excited by the light with a wavelength of 1550 nm. The Ge layer in Figure 2b was located on the top of the Si layer, thinned by etching. In each case, electric-field intensities were normalized by that of the dominant mode, i.e., the mode traveling along the edge of the disk. For this reason, faint modes showed a large difference in intensity from the dominant mode. As we expected in Type B, owing to the ridge waveguide, the high significant power was confined to the waveguide structure and was thus further influenced by the refractive-index change, thereby increasing the sensing ability.

When characterizing a disk-shaped resonator, loss analysis by a light-propagation pattern is important. Input light is coupled from the bus waveguide to the Si disk, in which the light has four propagation patterns: a circular trip inside the Si disk, coupling back to the bus waveguide, diffusion into the cladding material, and absorption by the Ge disk. The resonance characteristics are determined by the circular trip, and the other three propagation patterns are engaged in optical loss. The amount of light coupled back to the bus waveguide is determined by the coupling gap and plays a major role in determining the initial amount of light. Diffusion into the cladding material is determined by the index difference between the Si disk and the cladding material. Because light traveled from the Si disk to the Ge disk through evanescent coupling, light absorption into the Ge disk was mainly affected by the Ge thickness and the radius difference between the two disks. As mentioned before, the etch depth is an additional factor affecting the amount of diffused and absorbed light in Type B. Diffusion is a pure loss factor of the resonator, but absorption into the Ge disk is directly related to the responsivity of the photodetector.

## 3. Simulation Setup and Results

A three-dimensional simulation was conducted at a temperature of 300 K. All of the simulation boundaries were set as an eight-layered perfectly matched layer to prevent light reflecting back into the Ge disk. In the simulations, an optical broadband source (exiting TE fundamental mode) that had a wavelength span of 20 nm was used. This wavelength range was divided into 401 points to investigate the normalized light absorption at each wavelength. This corresponded to the experiment measurements by sweeping the wavelength of the tunable laser with a step of 50 pm.

To evaluate the effect of the recess for both structures, the power absorbed by the Ge layer was plotted as a function of wavelength for the performance of the Type A and B sensors according to the combination of geometric parameters, such as the Ge thickness (*T_Ge_*), and the radii (*R_Si_* and *R_Ge_*). The amount of normalized light absorption was plotted as a function of wavelength while sweeping the parameters. The light-absorption amount was normalized to the source power, which means that it showed a maximum value of 1 when the Ge disk absorbed all of the light power emitted from the optical source. The *R_Ge_* was set to be smaller than the *R_Si_*, and the inner gap (*G_in_*) between them was optimized to preserve a ridge waveguide forming along the edge of the bottom disk. The coupling gap was set to 100 nm for all simulations. The refractive index of the cladding was tested in the range from 1.31 to 1.35, which covers the refractive index of the NaCl solution over a concentration range from 0% to 25% and the refractive indices of some organic liquids (methanol, ethanol, and isopropyl alcohol) [2].

### 3.1. Inner-Gap (G_in_) Dependence

**Figure 4:**
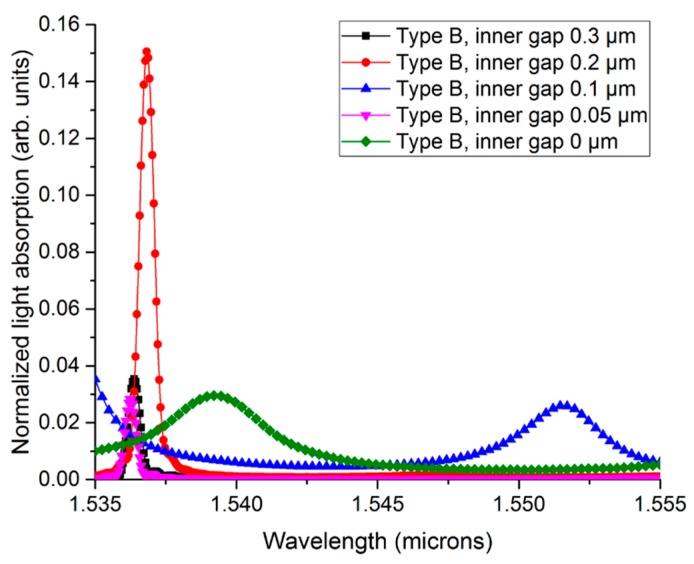
Normalized light absorption as a function of wavelength with *G_in_* from 0 to 0.5 μm in Type B.

Before investigating the performance of Types A and B, we had to optimize the inner-gap size to satisfy the waveguide condition in Type B. Figure 4 shows normalized light absorption as a function of wavelength for *G_in_* = 0 to 0.3 μm. For all cases, the *R_Si_*, *T_Ge_*, and *n_c_* were fixed to 3 μm, 300 nm, and 1.31, respectively.

From the figure, we can see that the *Q* value appeared to be 2500 or more at *G_in_* = 0.05, 0.2, and 0.3 μm. The results show the highest *Q* value to be at *G_in_* = 0.05. However, structures with such a narrow inner gap had very low light absorption levels (<10%). This is directly related to signal detection level. Also, the epitaxial growth of Ge on Si with a narrow gap could increase the fabrication complexity dramatically. Therefore, we chose the inner gap of 200 nm for further simulations of Type B.

### 3.2. Thickness (T_Ge_) Dependence

**Figure 5:**
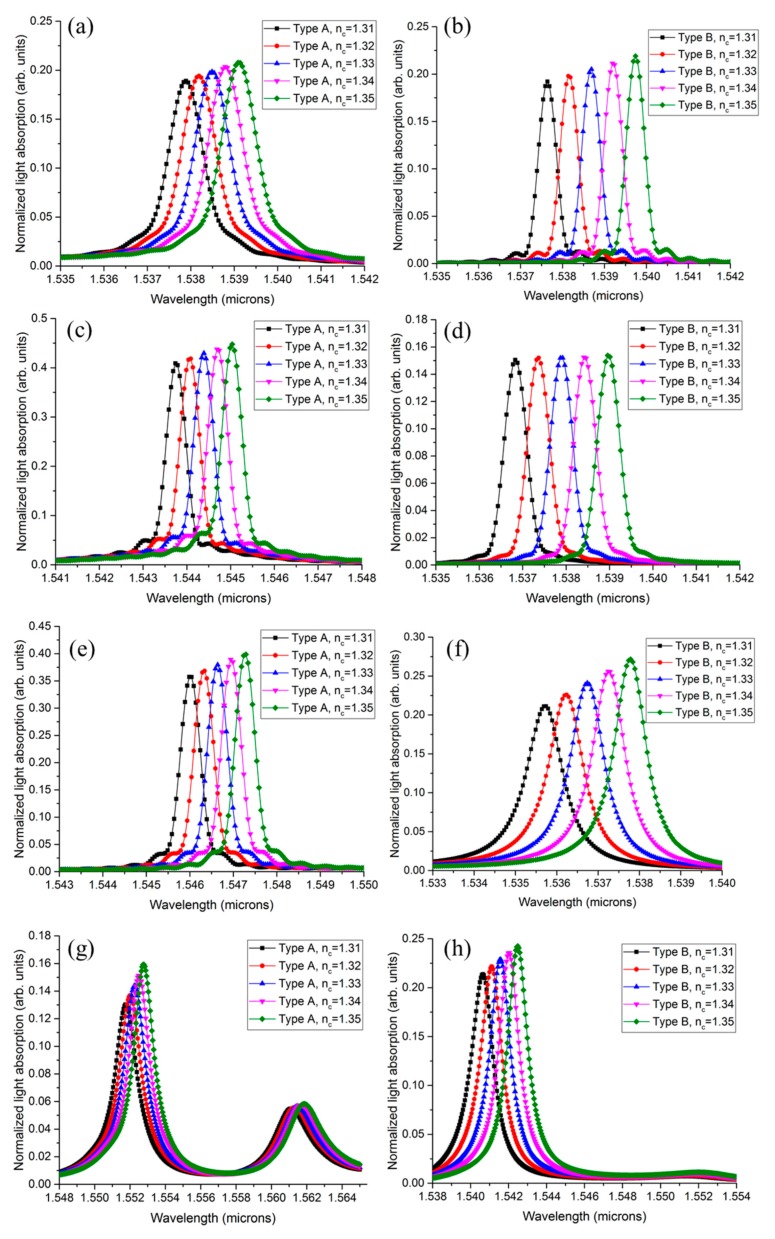
Graphs of normalized light absorption inside the Ge disk as wavelength function. Light absorption normalized to the optical-source power. Graphs plotted for Types A and B with different *T_Ge_*: (**a**,**b**) 200 nm; (**c**,**d**) 300 nm; (**e**,**f**) 400 nm; (**g**,**h**) 500 nm.

Figure 5 shows the normalized light absorption as a function of wavelength for *T_Ge_* = 200 to 500 nm in Types A and B. For each case, the *n_c_* was varied from 1.31 to 1.35 to determine the detectable sensing range and sensitivity, as well as the *Q*-factor. In the simulation, the *R_Si_*, *R_Ge_*, and *G_in_* were set to 3, 2.3, and 0.2 μm, respectively.

For the same *T_Ge_*, Types A and B showed different resonance characteristics owing to the recess. These are summarized in Table 1. For the same structure and *T_Ge_*, *Q* and *S* were slightly different with respect to the *n_c_* because the TE fraction of the mode was evanescently coupled to the Ge disk, owing to the change in the effective refractive index. Here, we took the mean values of *Q* and *S* when comparing the resonance characteristics. Type A showed a minimal *ILOD* of 1.66 × 10^−2^ RIU at a *T_Ge_* of 300 nm and 400 nm, but the light absorption was decreased at *T_Ge_* = 400 nm. Type A showed a sensitivity of 30 nm/RIU for all cases, except for that of 500 nm. The *Q* value of Type B became larger when *T_Ge_* decreased. Additionally, Type B showed a relatively small *ILOD* variation with *T_Ge_* compared to that of Type A because the area where the interaction between the light and the cladding material occurred was preserved by the ridge waveguide structure along the edge of the Si disk. The sensitivity of Type B was approximately 52.5 nm/RIU for all cases, except for that of 500 nm, but it was also reduced at *T_Ge_* = 500 nm, as with Type A. This was because the light confinement inside the ridge structure was weakened by the thick Ge layer, which had a higher refractive index than that of Si. The minimal *ILOD* of 0.95 nm/RIU was achieved in Type B for *T_Ge_* = 200 nm.

Sensitivity is mainly determined by the interaction area between the electric field of the propagating optical mode and the cladding material. Therefore, the thickness of the Ge disk did not significantly influence sensitivity. However, for *T_Ge_* > 500 nm, sensitivity decreased because the coupling was more dominant than the interaction with the cladding material. Additionally, we expected the couplings in Types A and B to be different owing to the recess [16,17]; thus, the *T_Ge_* corresponding to the maximal *Q* also appeared to be different.

### 3.3. Disk-Size Dependence

**Figure 6:**
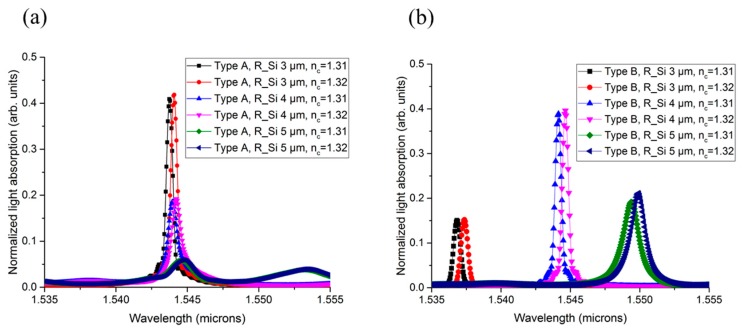
Simulation results by sweeping Si disk size from 3 to 5 μm for (**a**) Type A and (**b**) Type B.

The size of the contact metal formed on top of the Ge depends on the fabrication method and limitations. It is advantageous to make the sensor smaller for on-chip configuration. Figure 6 shows normalized light absorption for different disk sizes. The simulations were conducted for *R_Si_* = 3 to 5 μm. In consideration of the inner gap (0.2 μm) and the width (0.5 μm) of the waveguide, the *R_Ge_* was set to the value of the *R_Si_* − 0.7 μm for all cases. Additionally, the *T_Ge_* was fixed at 300 nm. For each structure, a simulation was executed for *n_c_* = 1.31 and 1.32.

As seen in Figure 6, both types of structure showed a red shift in resonant wavelength as the *R_Si_* increased. This peculiarity stood out in Type B because it had a confinement mechanism to the propagating mode. Additionally, the light-absorption amount was reduced with increasing *R_Si_* in Type A. Disk-size-dependent resonance characteristics with the *R_Si_* are summarized in Table 2. The error between the average of the actual values and mean values was under 4.8%. In Type A, the absorption peak, *Q*, and *S* were reduced at a larger *R_Si_*. On the contrary, Type B showed the best sensing performance at *R_Si_* = 4 μm.

In Type A, the propagating modes mainly existed under the Ge layer, i.e., near the center of the Si disk. In Type B, however, the propagating mode was confined to the center of the ridge waveguide. As the disk size increases, then, the length of the ring waveguide also increases. This explains the large shift in wavelength in Type B.

## 4. Discussion

The disk-shaped Ge-on-Si photodetector sensors showed a relatively low *Q* value, because light absorption acted as an additional loss factor. Thus, the *ILOD* of Type A was over 1.66 × 10^−2^ RIU for all analyzed cases. Type B was newly proposed in this work and showed sensitivity improvements by 20 nm/RIU compared with Type A for almost all cases of different combinations of geometric parameters. Moreover, Type B had a similar *Q* value to Type A, and it resulted in a lower *ILOD*. The sensing performance of the optical refractive-index sensors with different resonator types is summarized in Table 3. As shown in Table 3, we obtained a minimal *ILOD* of 0.95 × 10^−2^ with Type B. This value was relatively low compared with ring and disk resonator sensors with high *Q*. However, Type B had a higher sensitivity than that of the regular disk resonator with the same size. In addition, the *ILOD* of Type B was similar to that of the disk-shaped Si photodetector sensor with a disk radius larger by tenfold or more.

Thus, the simulation results confirm that the proposed recess structure increases the sensitivity of the sensing device and also enlarges the interaction area between the propagating optical mode in the disk and the cladding material. Additionally, the simulation performance of the disk with a 3 μm radius showed that a sensitivity of 52.5 nm/RIU, a Q-factor of ~3000, and an *ILOD* of 0.95 × 10^−2^ RIU can be achieved by optimizing geometric parameters such as disk thickness, radius, and size. In particular, it is expected that the advantage of using Ge is that it makes sensor devices smaller due to its high absorption coefficient and the combined design of the resonator and the photodetector. We also proposed a combined structure with a recess that improves sensitivity without enlarging sensor structure. Moreover, the photodetector-combined sensor array is easy to make, just like a photodetector array, because each sensor can be independently operated without any external optical receiver. Therefore, the proposed structure is useful in the application of LoC systems over other photodetector sensors [12,13].

## Figures and Tables

**Table 1:** Resonance characteristics according to structure type and Ge thickness (*T_Ge_*).

Structure Type	*T_Ge_* (nm)	Normalized Minimal Absorption Peak	Resonance Wavelength ^1^ (μm)	*Q* ^1^	*S*^1^ (nm/RIU)	*ILOD* (RIU)
Type A	200	0.189	1.53849	1538	30	3.33 × 10^−2^
	300	0.409	1.54439	2864	32.5	1.66 × 10^−2^
	400	0.357	1.54664	2812	32.5	1.69 × 10^−2^
	500	0.13	1.55227	1024	25	6.06 × 10^−2^
Type B	200	0.192	1.53868	3077	52.5	0.95 × 10^−2^
	300	0.15	1.5379	2549	53.25	1.13 × 10^−2^
	400	0.211	1.53675	1537	51.25	1.95 × 10^−2^
	500	0.214	1.54157	1139	46	2.9 × 10^−2^

^1^ Mean value. Note: *ILOD*, intrinsic limit of detection; RIU, Refractive Index Unit.

**Table 2:** Resonance characteristics according to structure type and *R_Si_*.

Structure Type	*R_Si_* (μm)	Normalized Minimal Absorption Peak	Resonance Wavelength ^1^ (μm)	*Q* ^1^	*S* (nm/RIU)	*ILOD* (RIU)
Type A	3	0.409	1.54392	2807	35	1.57 × 10^−2^
	4	0.186	1.54412	1873	25	3.3 × 10^−2^
	5	0.058	1.54464	736	20	10.5 × 10^−2^
Type B	3	0.15	1.5371	2584	54.5	1.09 × 10^−2^
	4	0.389	1.54439	2948	50	1.05 × 10^−2^
	5	0.192	1.54965	1141	50	2.72 × 10^−2^

^1^ Mean value.

**Table 3:** Sensing performance of optical refractive index sensors.

Sensor Type	*Q*	*S* (nm/RIU)	*ILOD* (10^−3^ RIU)	Structure Radius (μm)	Reference
Disk	38,000	28	1.45	3	[1]
Suspended disk	112	150	~91	0.8	[2]
Ring	15,000	150	~0.69	20	[7]
Disk-shaped Si photodetector	4400 ^1^	40 ^1^	8.3	40	[12]
Cascaded rings ^2^	15,000 to 25,000	>100	0.5	10 and 30	[14]
Disk-shaped Ge-on-Si photodetector	3077 ^1^	52.5 ^1^	9.5	3	This work

^1^ Value was calculated assuming that the sensing area was not covered by contact metal. ^2^ Sensor used two cascaded rings.
